# News and Events

**Published:** 2008

**Authors:** T. Ganesh

**Affiliations:** Senior Medical Physicist, Department of Radiation Oncology, King Fahad Specialist Hospital, Dammam-31444, Kingdom of Saudi Arabia. Email: sptgnadar@yahoo.com

## Granite Tops Raise Fear of Radiation

The Marble Institute of America is planning to develop a testing protocol for granite following more reports of hot or potentially hazardous granite countertops emitting radiation at higher-than-background levels. Few granite slabs have tested at levels 100 times or more above background.

From: The New York Times, July 24, 2008

## Incident Involving Radioactive Material at the International Atomic Energy Agency Safeguards Laboratory

Pressure build-up in a small sealed sample bottle in a storage safe resulted in plutonium contamination of a storage room on August 3, 2008 at the International Atomic Energy Agency's (IAEA) Safeguards Analytical Laboratory in Seibersdorf. The Laboratory is equipped with multiple safety systems to prevent the release of radioactivity to the environment. The laboratory routinely analyses small samples of nuclear material (uranium or plutonium) as part of the IAEA´s safeguards verification work.

From: IAEA Press Release dated August 3, 2008

## Overexposure During Co-60 Source Exchange

Two radiation workers, while working on a source exchange of a decayed cobalt-60 source in a hospital in the State of São Paulo, Brazil on July 21, 2008, received significant overexposure. With the removal tool, the worker transferred the source drawer from the unit head to the transfer cask. However, after this transfer, the tool was not completely disengaged from the source drawer with the result when the tool was pulled out, the source drawer momentarily came out of the transfer cask before the worker pushed it back.

Based on an emergency thermo-luminescent dosimeter (TLD) reading, one of the workers had received a whole body equivalent of 135 mSv and the other worker received 0.7 mSv.

On August 15, 2008, U.S. regulatory authorities learned that one of the fingers of the individual who received a high whole body dose was blistering. Based on the physical response, the company's medical consultant now estimates the extremity dose to be approximately 25 Sv.

From: http://www-news.iaea.org/news/default.asp

## Vertical Scanning Nanobeam

The Surrey Ion Beam Centre at the University of Surrey has a unique proton beam facility: a 10-nanometre diameter vertical scanning beam of protons to scan individual cells, the world's first such facility. The new beamline will help understand how radiation affects living cells and show reactions between drugs and radiation. Its proton nanobeam is capable of scanning 100,000 cells per hour and, using experimental data, computational research will construct virtual tumors and devise new treatment strategies. Scientists are looking forward to some significant discoveries.

From: The Guardian, Thursday, August 21, 2008

## The World's Largest and Highest-Energy Particle Accelerator

The world's largest particle collider passed its first major tests by firing two beams of protons in opposite directions around a 17-mile (27-km) underground ring (underneath the Franco-Swiss border between the Jura Mountains and the Alps near Geneva, Switzerland) on September 10, 2008 in what scientists hope is the next great step to understanding the makeup of the universe. After a series of trial runs, two white dots flashed on a computer screen indicating that the protons had travelled clockwise along the full length of the 4 billion Swiss franc ($3.8 billion) Large Hadron Collider (LHC)—described as the biggest physics experiment in history. Physicists around the world now have much greater power to smash the components of atoms together in attempts to learn about their structure. Eventually, two beams will be fired at the same time in opposite directions with the aim of recreating conditions a split second after the Big Bang, which scientists theorize was the massive explosion that created the universe. Scientists hope to eventually send two beams of protons through two tubes about the width of fire hoses, speeding through a vacuum that is colder and emptier than outer space. The paths of these beams will cross, and a few protons will collide. The collider's two largest detectors—essentially huge digital cameras weighing thousands of tons—are capable of taking millions of snapshots a second. The CERN (European Organization for Nuclear Research) experiments could reveal more about dark matter, anti-matter, and possibly the hidden dimensions of space and time. It could also find evidence of the hypothetical particle, the Higgs boson, which is sometimes called the God particle because it is believed to give mass to all other particles, and thus to matter that makes up the universe.

This important scientific event had significant Indian contributions. A 30-member Indian team chosen by the Department of Atomic Energy's Variable Energy Cyclotron Centre in Kolkata developed one of the Photon Multiplicity Detectors (PMDs) used in the LHC. There were 15 PMDs that will recreate, in the current experiment, conditions that supposedly existed just after the Big Bang. The scientists came from the faculties of the Rajasthan University, Jammu University, Punjab University, IIT Mumbai, and the Institute of Physics, Bhubaneshwar.

Nine days after the major achievement on September 10, 2008, the LHC developed snags in the form of large helium leak and is currently undergoing repairs. The accelerator complex will not start its experiments until the middle of next year. LHC beams will then follow.

From: The Hindu dated September 11, 2008; http://press.web.cern.ch/press/PressReleases/Releases2008/PR10.08E.html and http://www.timesonline.co.uk dated November 17, 2008

## Overexposures Involving Ir-192 Industrial Radiography Cameras

Three separate incidents involving Ir-192 industrial radiography cameras at different locations resulted in overexposures of workers ranging from whole body doses of 2 mSv to 160 mSv. The dose to extremity was as high as 2.36 Sv for one of the workers. The incidents happened in Ardmore, Oklahoma, U.S.; Tarragona, Spain; and Houston, Texas, U.S. during September 2008.

From: http://www-news.iaea.org/news/default.asp

## Academics Fear Nuclear Legacy

Officials at Manchester University are struggling to contain rising concern among staff and students who have worked in the Rutherford Building since the mid-1970s. Four of their colleagues have died of cancer, and others fear their health may have been seriously damaged by materials left over from Ernest Rutherford's nuclear experiments. However, university officials maintain there is no risk to anyone in the building and stated there was not enough evidence yet to suggest a link between the deaths of former staff and possible exposure to radioactive contamination.

From: The Guardian, issues dated September 9, September 30 and October 21, 2008

## Swiss Pediatric Computed Tomography (CT) Survey Leads to National Dose Standards

Hospitals in Switzerland have uniformly adopted a set of dose reference levels (DRLs) for pediatric CT that are a function of age and weight of the patient. The move was the result of a national survey conducted by the University of Lausanne researchers who discovered disturbing variations in pediatric CT dose at different hospitals in the country. Radiation dose with respect to the DRL is verified at each hospital by inspectors of the Federal Office for Public Health when CT units are audited. In the near future, this will also be done by the medical physicists of each hospital. The University Institute for Radiation Physics is currently working on a project to standardize radiation dose for cardiac CT examinations for both children and young adults. The Institute welcomes international collaboration on this project.

From: www.AuntMinnie.com dated October 23, 2008

## Contaminated Lift Buttons

On October 8, 2008, France's Nuclear Safety Authority (ASN) found that 20 workers who handled lift buttons for supplying to a popular elevator manufacturer were exposed to radiation doses ranging from 1 to 3 mSv, which is more than the annual limit for the general public. The ASN said it had classified the incident as Level two on the international nuclear event scale. The French agency contacted the regulatory Authorities of the other countries involved including Sweden and India in order to exchange information on this event.

The safety agency said the buttons contained traces of radioactive cobalt-60. It was unclear where the contaminated scrap originated, although the metal was traced to a foundry in the western state Maharashtra in India.

From: http://www-news.iaea.org/news/default.asp and guardian.co.uk, October 24, 2008

## Recent Report of Radiotherapy Incident in Canada

A relocation of an orthovoltage treatment unit from one campus to the other in The Ottawa Hospital Cancer Center in the Fall of 2004, was followed by an error at the time of re-commissioning. The error was reflected as incorrect output tables for all 4 beam qualities and for all field sizes other than 10 × 10 cm^2^. A total of 1,019 patient treatments (palliative and curative) were delivered to 620 patients using the incorrect orthovoltage output tables during November 2004 to November 2007. Patients were under-dosed and the maximum under dosage received by the curative patient group was 17%. There were no reported over-dosages of patients.

During the incident review (Root Cause Analysis), the Panel of Experts confirmed that the error was due to the omission of a backscatter conversion factor for all fields other than 10 × 10 cm^2^. In the opinion of the Panel, a significant contributory factor was the extreme resource limitations experienced by the hospital's medical physics staff at the time of relocation. The criticality of medical physics for the safety and quality of highly specialized cancer treatments employing large doses of radiation appears not to have been adequately recognized.

The recommendations from the Panel of Experts focus on adoption, adherence and monitoring of specific nationally recognized protocols for commissioning, and quality assurance of radiation treatment equipment including orthovoltage units. Attention should also be paid to matching the scope and intensity of radiation therapy equipment installation and upgrading to available medical physics staffing.

From: www.iaea.org

## Syllabus Developed for Radiation Oncologists

To address the critical shortage of trained radiotherapy staff in developing nations, the International Atomic Energy Agency's (IAEA) Division of Human Health (NAHU) has developed a syllabus outlining the organization of training for radiation oncologists and the curriculum of subjects to be taught. The syllabus will be available to Member States and radiation oncologists in developing countries in 2009. This is just one in a series of IAEA produced syllabi for the training of professionals like medical physicists, radiation therapy technologists, and nurses.

The current course materials, which are aimed at doctors and radiation oncology specialists, include a full core curriculum, training objectives, and skill levels required, as well as a checklist for the effective auditing of radiation oncology training programs.

From: News Centre, www.iaea.org – dated November 12, 2008

## Recent Publications of Interest from the International Atomic Energy Agency (IAEA)

### Clinical Applications of SPECT/CT: New Hybrid Nuclear Medicine Imaging System

IAEA TECDOC Series No. 1597

This technical document (TECDOC) presents an overview of the SPECT/CT technology for use by nuclear medicine physicians, radiologists, and clinical practitioners. The publication also covers the current medical status of SPECT/CT imaging, the role of this technology in the clinical management of patients, and possible trends for future development.

http://www-pub.iaea.org/MTCD/publications/PDF/TE_1597_Web.pdf

### Cyclotron Produced Radionuclides: Principles and Practice

Technical Reports Series No. 465

This book provides a comprehensive treatment of cyclotrons, with a special emphasis on the production of radionuclides. This book will appeal to scientists and technologists interested in translating cyclotron technology into practice, as well as postgraduate students in this field.

http://www-pub.iaea.org/MTCD/publications/PDF/trs465_web.pdf

### Operational Guidance on Hospital Radiopharmacy: A Safe and Effective Approach

Clinically safe, effective, and economic practices in the area of hospital radiopharmacy can strengthen the overall performance of nuclear medicine services. This guidance provides practical points at different levels of operation including staff training, facilities, radiopharmaceutical practices, record keeping, and quality control.

http://www-pub.iaea.org/MTCD/publications/PDF/Pub1342_web.pdf

### Transition from 2-D Radiotherapy to 3-D Conformal and Intensity Modulated Radiotherapy

IAEA TECDOC Series No. 1588

This publication is intended to be a guide for radiotherapy centers making the transition from 2-D radiotherapy to 3-D conformal to intensity modulated radiation therapy (IMRT) and takes into account training, equipment, and other considerations necessary for the safe installation of a modern radiation oncology program.

http://www-pub.iaea.org/MTCD/publications/PDF/TE_1588_web.pdf

### Technetium-99m Radiopharmaceuticals: Manufacture of Kits

Technical Reports Series No. 466

This report describes the procedures for preparing 23 selected Tc-99m radiopharmaceutical kits. Details of the preparation of ten of the active ingredients are also included. This report is expected to serve as a guide to radiopharmaceutical manufacturing centers and centralized pharmacies involved in the production of kits. It will be a useful resource for the many hospital radiopharmacies that routinely use the kits to compound Tc-99m radiopharmaceuticals.

http://www-pub.iaea.org/MTCD/publications/PDF/trs466_web.pdf

### International

**1. Radiobiology - Endorsed by The European Society for Therapeutic Radiology and Oncology**
  Meeting Dates: March 22–27, 2009, Sydney, Australia
  For more details, send email to: max@conferencefocus.com.au.
**2. VI Symposium on Medical Physics and IV International Symposium on Medical Physics**
  Meeting Dates: June 15–18, 2009, Beskid Mountains, Poland
  Last date for submission of abstract: December 30, 2008
  For more details, log onto: http://www.ismp09.us.edu.pl/
**3. American Association of Physicists in Medicine (AAPM) 51st Annual Meeting**
  Meeting Dates: July 26–30, 2009, Anaheim, California
  Last date for submission of abstract: March 4, 2009
  For more details, log onto: http://aapm.org/meetings/09AM/

